# Editorial: Dietary polyphenols, gut microbiota, and human health

**DOI:** 10.3389/fphar.2022.1131074

**Published:** 2023-01-11

**Authors:** Lei Xu, Xin Zhang

**Affiliations:** Department of Food Science and Engineering, Ningbo University, Ningbo, China

**Keywords:** dietary polyphenols, gut microbiota, human health, COVID-19, ASD, DKD, IBD, liver

Dietary supplementation with polyphenols has multiple benefits for human health, and in particular, intake of dietary polyphenols has a significant positive impact on intestinal health and intestinal flora balance. This Research Topic provides a theoretical basis and reference for the scientific and adequate use of polyphenols for the prevention and treatment of intestinal diseases and the protection of intestinal health.

Coronavirus disease 2019 (COVID-19) is now spreading severely worldwide. In addition to the usual fever and severe respiratory symptoms, some patients with new coronavirus pneumonia also develop gastrointestinal symptoms. (Xu et al.) summarized that the patients infected with SARS-Cov2 had a stunted intestinal flora with decreased levels of *Lactobacilli* and *Bifidobacteria*, while the abundance of *Bacteroides massiliensis* and *Bacteroides ovatus* was negatively correlated with disease severity. Entry into cells *via* ACE2 receptors is a way for SARS-CoV-2 virus to invade the organism, and ACE2 is widely expressed in intestinal tissues. Probiotics can effectively regulate the intestinal flora of patients with pulmonary infections, establish a normal commensal flora, reduce the invasion of potentially pathogenic microorganisms into the intestine, and reduce the local inflammatory response and the occurrence of enterogenic diseases. Therefore, regulating the lung flora by regulating the intestinal flora may improve the immunity of the body, which in turn improves the symptoms of pneumonia and achieves therapeutic effects.

Autism spectrum disorder (ASD) begins in infants and young children, and is caused by genetic and environmental factors. Compared to healthy children, children with ASD are prone to co-morbid gastrointestinal symptoms, which may be associated with imbalance of intestinal flora. Short-chain fatty acids (SCFA) play a critical role in the occurrence and development of ASD. (Deng et al.) found that children with ASD were more likely to exhibit gastrointestinal problems and somewhat higher levels of propionic, butyric, and valeric acid levels, as well as higher levels of social impairment and poor sleep habits, compared to typically developing children. Although ASD may not directly lead to increased gastrointestinal symptoms, altered metabolites (e.g., SCFAs) may contribute to gastrointestinal symptoms. Research on the relationship between gut flora and the development of ASD and its specific mechanisms of action is still in its infancy, and therefore a large number of questions remain to be explored.

Diabetic kidney disease (DKD) is a common clinical chronic metabolic disease in world. Patients generally have moderate dysbiosis, with reduced abundance of butyric acid-producing bacteria and increased opportunistic pathogenic bacteria, which promote increased oxidative stress and sulfate reduction. Butyrate has been shown to promote the secretion of glucagon-like peptides GLP-1 and PYY from L cells in the colon through G protein-coupled receptor binding. (Cheng et al.) found that butyrate acts as an inhibitor of histone deacetylase *in vivo* and acts as an epigenetic regulator by upregulating miRNAs or inducing histone butyrylation and autophagy processes. Therefore, further investigation of the action of butyrate in DKD prevention is warranted.

Inflammatory bowel disease (IBD) can be divided into ulcerative colitis and Crohn’s disease, which is a chronic inflammatory disease of the gastrointestinal tract. *Bifidobacteria*, as the dominant beneficial flora in the intestinal tract, adhere to the intestine by themselves or through their metabolites, preventing the colonization and translocation of pathogenic bacteria. (Bi et al.) found that long-term oral administration of *B. longum-Endo* was effective against tumors, and the abundance of *Lactobacillus*, *Bifidobacterium* and *Parabateroides* increased, while the abundance of some pathogenic bacteria (such as *Desulfovibrio* and *Enterobacter*) decreased. Therefore, the use of oral recombinant *B. longum-Endo* strains for the treatment of IBD is established, and the deeper excavation of whether the acting bacterial components and metabolites can target key proteins that inhibit the inflammatory pathway is significant for future clinical treatment.

Intestinal flora imbalance is common in patients with type 2 diabetes (T2D), and the pattern of imbalance is mainly characterized by a decrease in butyrate-producing bacteria and an increase in opportunistic pathogenic bacteria. (Costabile et al.) provided mice with a high-fat, high-fructose diet and found a decrease in the abundance of lactic acid bacteria and an increase in the abundance of proteobacterial taxa (especially Oxalobacteraceae) and Lachnospiraceae, and this microbial composition (regulated by *in vitro* fermentation of whole grains) had an effect on the secretory capacity of pancreatic β-cells. Since dysbiosis of intestinal flora is an important initiating factor of glucolipid metabolism disorder in T2D, the adjustment of intestinal flora can be used as a therapeutic target based on glucose lowering to improve insulin resistance and glucolipid metabolism disorder in patients and delay the disease progression.

Primary liver cancer is one of the most important malignant tumors threatening human health in the world. The dysbiosis of intestinal flora can lead to multiple pathways driving the development and progression of hepatocellular carcinoma, therefore improving intestinal flora can play a role in modifying or even reversing the development of hepatocellular carcinoma. (Li et al.) found that Jiawei Xiaoyao San can restore the composition of intestinal flora by regulating the abundance of probiotics, and play a role in the treatment of liver cancer through overall regulation. However, the specific mechanism of how it can play a role in liver cancer treatment by regulating intestinal flora has not been reported yet and still needs to be studied in depth.

A complex network of interactions existed between diet, intestinal flora and human health, and future research will further reveal the key role of diet in this regard with the continuous advancement of histological techniques and immunological research tools. However, further research is needed in the future to discover unknown polyphenol microbial metabolites and to correlate specific gut microbes with microbial metabolites.

